# Complex Hydrogels Composed of Chitosan with Ring-opened Polyvinyl Pyrrolidone as a Gastroretentive Drug Dosage Form to Enhance the Bioavailability of Bisphosphonates

**DOI:** 10.1038/s41598-018-26432-2

**Published:** 2018-05-25

**Authors:** Chia-Yu Su, Hsiu-O Ho, Ying-Chen Chen, Yu-Ting Yu, Der-Zen Liu, Fang-Ching Chao, Ming-Thau Sheu

**Affiliations:** 1 School of Pharmacy, College of Pharmacy, Taipei Medical University, Taipei, Taiwan, ROC; 2 Graduate Institute of Biomedical Materials and Tissue Engineering, College of Biomedical Engineering, Taipei Medical University, Taipei, Taiwan, ROC; 3 Clinical Research Center and Traditional Herbal Medicine Research Center, Taipei Medical University Hospital, Taipei, Taiwan, ROC

## Abstract

Complex hydrogels formed with chitosan (CS) and ring-opened polyvinyl pyrrolidone (*ro*PVP) as a swellable mucoadhesive gastroretentive drug dosage form (*sm*GRDDF) were prepared and characterized. CS/*ro*PVP hydrogels were produced by blending CS with *ro*PVP obtained by basic treatment of PVP. Effects of the heating time and NaOH concentration employed for preparing *ro*PVP, and CS molecular weights (Mws), and *ro*PVP/CS ratios on the swelling ability of the resultant hydrogels were characterized. Rheological characteristics were further examined. Results demonstrated that *ro*PVP obtained in a 0.5 M NaOH solution heated to 50 °C for 4 h was suitable for producing complex hydrogels with CS. At a *ro*PVP/CS ratio of 20:1, hydrogels composed of three different Mws of CS possessed optimal swelling and mucoadhesive abilities and rheological properties. *In vitro* dissolution revealed sustained drug release. A pharmacokinetic study exhibited that the plasma profile of alendronate followed a sustained manner with 3-fold enhancement of the oral bioavailability. In conclusion, the *sm*GRDDF composed of CS/*ro*PVP complex hydrogels was successfully developed and is potentially applicable to improve the clinical efficacy of bisphosphonates.

## Introduction

Osteoporosis is a commonly occurring disease during the aging process. Currently, the drug of choice for treating this disease is bisphosphonates. However, local irritation of the mucous layer of the upper digestive tract and very low oral bioavailability (BA) are their limitations^1^. For those drugs mainly absorbed in the proximal part of the gastrointestinal (GI) tract, a short transit period (of less than 6 h in the stomach and upper small intestine) was expected to have a short absorption phase, that is often accompanied by lower BA^2–4^. Gastroretentive drug dosage forms (GRDDFs) with a longer gastric residence time would potentially be desirable to achieve therapeutic benefits of drugs that are mainly absorbed in the proximal part of the GI tract. After oral administration, drug-loaded GRDDFs could be retained in the stomach so that they release the drug there in a sustained manner, allowing the drug to be continually supplied to its absorption site^5^. This mode of administration would prolong the period in which serum drug concentrations are within “therapeutic levels”.

There are three practically applicable designs of GRDDFs: (1) mucoadhesion to the gastric mucosa to extend the residence of GRDDFs in the stomach; (2) density modification (floating or sinking) to make the dosage form float or sink and prevent it from leaving the stomach due to limited access to the pylorus; and (3) expansion (swelling) of a GRDDF to a size that is too large to pass through the pylorus, thus prolonging gastric retention^6^. Various combined gastroretentive mechanisms have also been reported to enhance gastroretentive capabilities^7–9^. Although many studies have attempted to develop GRDDFs, a combined mechanism of swelling with either floating or mucoadhesion seems to offer greater safety and efficiency for clinical uses^10^. We previously reported the combination of hydroxyethyl cellulose (HEC) and sodium carboxymethyl cellulose to improve the extent of swelling and floating of GRDDFs for losartan^11^, and also the combination of HEC, chitosan (CS), and sodium bicarbonate^12^.

Hydrogels are polymeric materials with a three-dimensional (3D) network structure capable of enormous swelling in aqueous media. Hydrogels are called superporous hydrogels when they present pores of a size of hundreds of micrometers, which can absorb a great amount of water in a very short time. The high swelling kinetics lead to favorable development as gastroretentive devices for controlled release^13^. In their previous work, Chen *et al*. found that to be used as an effective gastroretentive device, hydrogels should possess rapid swelling and also be biocompatible and biodegradable, with a high swelling capacity and high mechanical strength, and be stable in an acidic condition of pH 1.2^14^. Therefore, CS has garnered considerable attention in pharmaceutical research^15^, since CS is a swellable hydrogel in acidic media, and is biocompatible, biodegradable, and nontoxic. Further, CS has been explored as a vehicle for gastric drug delivery due to its excellent mucoadhesive properties^16–18^. However, CS matrices are known to be fragile and exhibit uncontrollable porosity. The use of pure CS formulations in oral administration is also limited due to their fast dissolution in the stomach and their limited capacity for controlling the release of drugs.

CS gels with improved mechanical strength can be formed by chemical cross-linking with a cross-linker such as glutaraldehyde^19,20^ and glyoxal^21^ or physical cross-linking as ionically cross-linked CS with different multivalent phosphates, namely pyrophosphate (Pyro) and tripolyphosphate (TPP)^22^ and in polyionic complexes of positively charged CS with a negatively charged natural polymer like alginate or a synthetic one like polylactic acid. These chemically or physically cross-linked CS hydrogels not only swell but also have intragastric-floating characteristics that prolong retention of the GRDDF in the stomach. With these advantages, CS hydrogels have been widely exploited for GRDDFs^23–26^. However, chemical cross-linking agents may be toxic and have other undesirable environmental or manufacturing problems. Also, the decreasing extent of amino groups in CS after cross-linking reduces the mucoadhesive capability of CS hydrogels. Physically cross-linked hydrogels are more biocompatible due to the lack of chemical cross-linkers and are well tolerated compared to covalent systems^27^. Between two types of physically cross-linked hydrogels, polyionic complexes of CS seem to possess an optimal swelling ratio with only slightly reduced mechanical strength compared to that of ionically cross-linked CS.

A combination of CS:polyvinyl pyrrolidone (PVP) at a weight ratio of 2:1 forms a brittle solid in a dry state that swells rapidly (<3 h) and extensively (110~115-fold) in acidic water (pH 2.0) without dissolving as illustrated in US patent 6730327 by Zentner *et al*.^28^ However, exposure of this combination to pH 2.0 for more than 4 h resulted in substantial dissolution. Nevertheless, PVP only with a desired quantity of acidic groups expressed by opened lactam rings blended with CS formed a stable hydrophilic gel which absorbed water without dissolving or disintegrating^29^. Further, Suknuntha *et al*. reported that the mucoadhesion of CS could also be enhanced by blending it with PVP^30^. PVP was demonstrated to control the porous nature of the CS-PVP blend as well, and it exhibited controlled drug release from freeze-dried matrices, making CS-PVP freeze-dried matrices a potential candidate for the controlled release of antibiotics in the stomach’s acidic environment^31^.

In the present study, polyionic complex hydrogels of CS with ring-opened (*ro*)PVP possessing a combination of mucoadhesive and swelling capabilities to prolong gastric retention times of alendronate in the upper part of the GI tract with a sustained drug release pattern were developed and characterized. While bioadhesion ensures that the dosage form adheres to the gastric mucosa, rapid and high degrees of swelling help delay clearance through the pyloric sphincter. Further, the mucosal adhesion of CS and the higher viscosity and rigidity of the complex hydrogel potentially minimize local irritation by the active ingredient (alendronate sodium) by forming a protective layer on the mucosal membrane of the stomach and by the slower release of the active ingredients^32^. Also, positive and negative charge interactions between alendronate sodium and CS decrease irritation of the upper digestive mucous. With these superior properties for such a novel swellable mucoadhesive (*sm*)GRDDF system, the active ingredient (alendronate sodium) with an absorption window located in the upper GI tract is expected to be continually and sustainably released upstream of the stomach and passed through the absorption region in the upper GI tract resulting in enhanced BA.

## Results

### Effect of Heating Times on the Generation of *ro*PVP to Form CS/*ro*PVP Complex Hydrogels

Results shown in Figure S1A (Supplemental file) demonstrate that without heating treatment of PVP, the addition of CS of any Mw did not form CS/*ro*PVP hydrogels, leading to the least extent of swelling for the obtained tablets in simulated gastric acid after being soaked in the solution for 6 h. When heating treatment of PVP was increased to 8 h at 100 °C, the swelling of complex hydrogels formed with low-Mw (LMw) CS only lasted for 2 h and then began to dissolve in simulated gastric acid, while swelling of complex hydrogels formed with both medium-Mw (MMw) and high-Mw (HMw) CS maintained their diameter at 12 h with a 1.8-fold increase without dissolution. Contents of carboxylic groups in the *ro*PVP with different heating time treatments (0, 1, and 8 h) at 100 °C were 8.82, 9.24, and 17.64 mmole, respectively. It was concluded that a desired heating time at elevated temperature for the basic treatment of PVP to generate a desired amount of *ro*PVP is required to form complex hydrogels with a desired strength.

### Effects of NaOH Concentrations on the Generation of *ro*PVP for the Formation of CS/*ro*PVP Complex Hydrogels

Effects of NaOH concentrations on the generation of *ro*PVP to form complex hydrogels with HMw CS were examined at a heating temperature of 50 °C for 4 h. Contents of carboxylic groups in *ro*PVP with different NaOH concentration treatment (0, 0.5, 1, and 1.5 M) were 8.82, 30.17, 62.49, and 79.27 mmole, respectively. Figure S1B (Supplemetal file) shows that the swelling of complex hydrogels formed with HMw CS and *ro*PVP generated by heating treatment of PVP in a 0.5 M NaOH solution was found to be optimal. Those tablets could remain in a swollen state for at least 12 h with a 1.2-fold increase in diameter and no sign of dissolution. In contrast, complex hydrogels formed with HMw CS and *ro*PVP generated by treatment at two other higher NaOH concentrations (1.0 and 1.5 M) showed a fast swelling rate in the beginning, but all those hydrogel tablets stopped swelling at 5 h, and then the swollen tablets gradually dissolved until they had completely disappeared.

### Effects of the CS Mw and *ro*PVP/CS Ratio on the Formation of CS/*ro*PVP Complex Hydrogels

The effects of the CS Mw and *ro*PVP/CS ratio on the mechanical properties of CS/*ro*PVP complex hydrogels so obtained were evaluated based on the rheological characteristics as follows.

#### Oscillatory Amplitude Sweep

**Figure 1:**
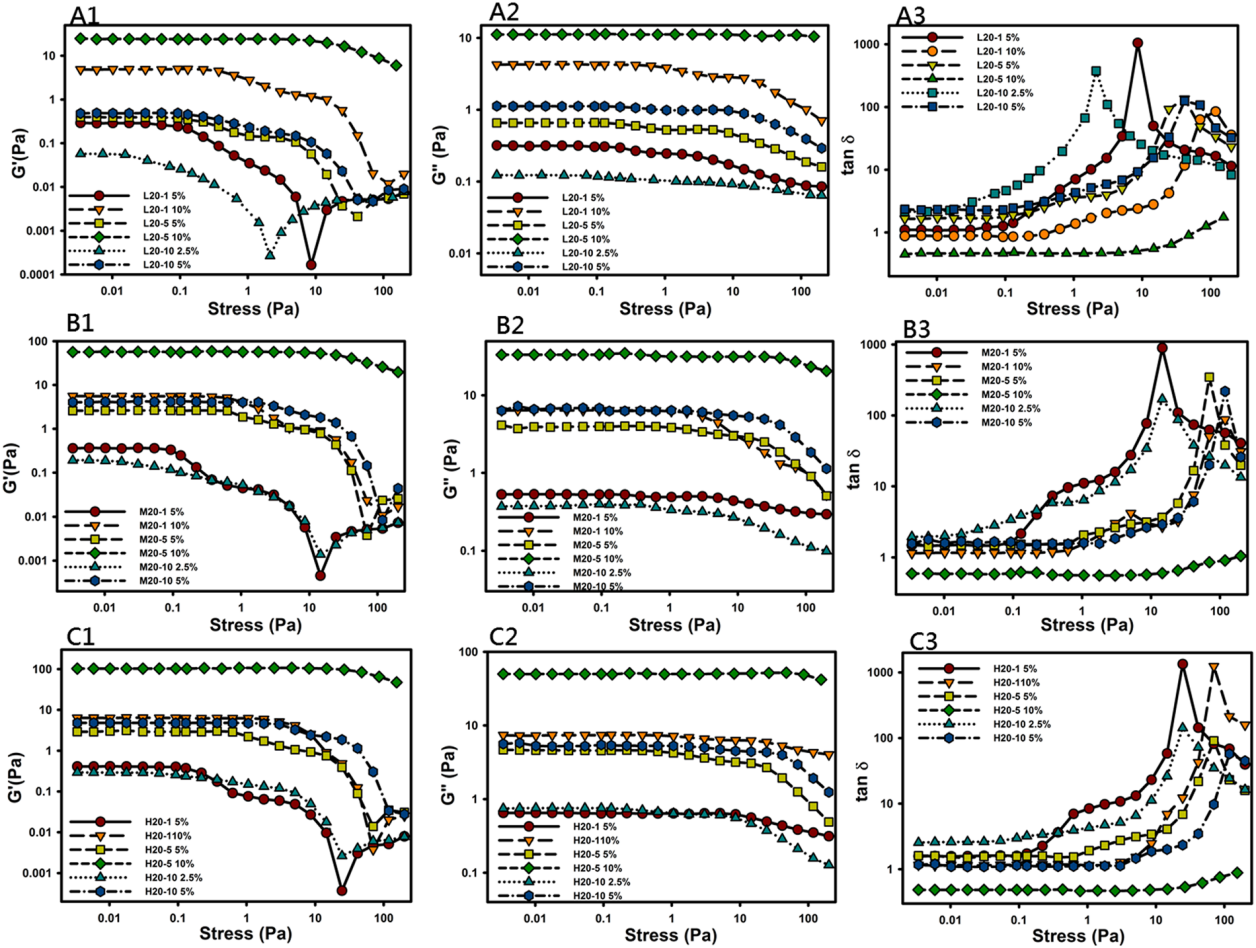
Comparison of the amplitude sweeps of ring-opened polyvinyl pyrrolidone (*ro*PVP)/chitosan (CS) hydrogels composed of various ratios (20:1, 20:5, and 20:10) of *ro*PVP/CS and different molecular weights (Mws) of CS (L, low Mw; M, medium Mw; and H, high Mw). (**A1**) L20-1; (**A2**) L20-5; (**A3**) L20-10; (**B1**) M20-1; (**B2**) M20-5; (**B3**) M20-10; (**C1**) H20-1; (**C2**) H20-5; (**C3**) H20-10.

Figure 1 shows the effect of the stress amplitude on the storage modulus (G′, Fig. 1A1,B1 and C1), loss modulus (G″, Fig. 1A2,B2 and C2), and tanδ (Fig. 1A3,B3 and C3) of complex hydrogels. Figure 1A1,B1 and C1 demonstrate that increasing the polymer concentration (2.5%, 5.0%, and 10%), Mw of CS (LMw, MMw, and HMw), and the ratio of CS to *ro*PVP (1:20, 5:20, and 10:20) extended the linear viscosity region (LVR) in the plot of the storage modulus (G′) versus the stress amplitude for all complex hydrogels examined. In the LVR, the network structure of the hydrogels was maintained, and the applied stress did not influence the structural integrity, but showed linear viscoelastic behavior of a constant storage modulus with respect to the shear stress. Beyond the LVR, the storage modulus abruptly or gradually decreased, indicating that breakdown of the network structure of the hydrogels occurred as a consequence of the large deformation imposed. The stress at which a hydrogel begins to show nonlinear viscoelastic behavior is generally designated the critical shear stress. The higher the critical shear stress is, the higher the gel strength would be. It was expected that swollen hydrogels with a higher gel strength would be able to withstand the shear stress of the stomach by maintaining a larger swollen size of hydrogels than the opening of the ostium pyloricum to remain in the stomach for a longer duration. Therefore, complex hydrogels composed of HMw CS and a higher ratio of CS to *ro*PVP would be the preferable selection for formulating a GRDDF of swollen complex hydrogels with as high gel strengths as possible.

In plots of tanδ versus stress in Fig. 1A3,B3 and C3, only those hydrogels with a 10% concentration and a *ro*PVP/CS ratio of 20:5 showed tanδ values of <1, indicating that those hydrogels with G′ > G″ had gel viscoelastic behaviors dominated by elasticity. It also implies that for those complex hydrogels with a *ro*PVP/CS ratio of 20:5, a hydrogel concentration of 7.5~10% was desirable to form a solid-like hydrogel with a higher gel strength. For those complex hydrogels with a *ro*PVP/CS ratio of 20:1 or 20:10, although their tanδ values were >1, which indicated gel viscoelastic behavior dominated by viscosity, values closer to 1 with an increasing complex hydrogel concentration were observed, indicating a gradual transformation from a viscoelastic liquid to a viscoelastic solid at an increasing concentration.

**Figure 2:**
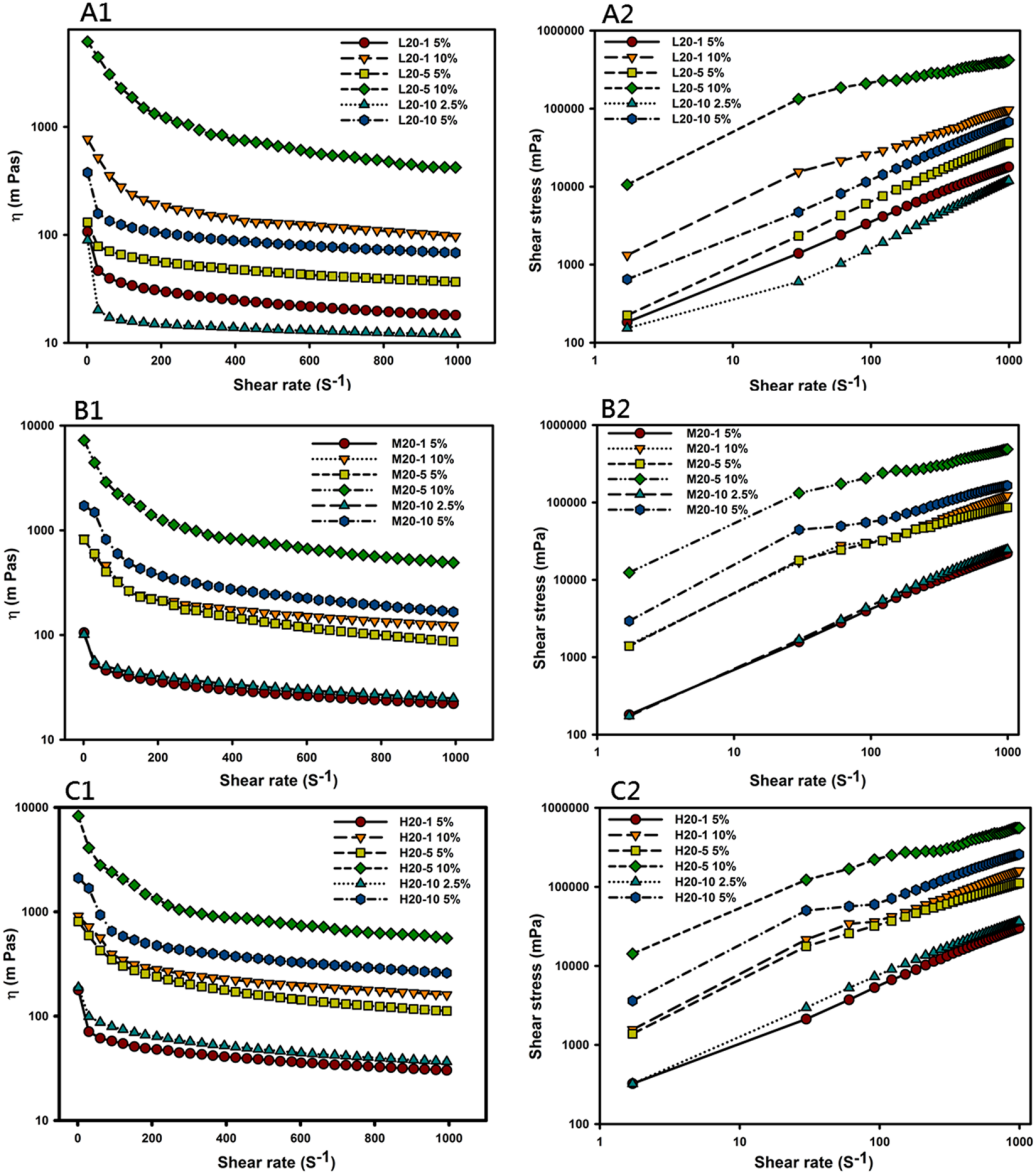
Dynamic viscosity (η) versus the shear rate ($\dot{\gamma }$) (**A1**,**B1** and **C1**) or log shear stress (τ) versus the log shear rate ($\dot{\gamma }$) (**A2**,**B2** and **C2**) for ring-opened polyvinyl pyrrolidone (*ro*PVP)/chitosan (CS) hydrogels composed of *ro*PVP complexed with different molecular weights (Mws) of CS ((**A1** and **A2**) low Mw, (**B1** and **B2**) medium Mw, (**C1** and **C2**) high Mw) at three *ro*PVP:CS ratios of 20:1, 20:5, and 20:10.

Since most of these complex hydrogels were characterized as being a viscoelastic fluid with some extent of elastic solids, their flow behavior was measured, and results are illustrated by showing the viscosity (η, Fig. 2A1,B1 and C1) or shear stress (τ, Fig. 2A2,B2 and C2) versus the shear rate ($\dot{\gamma }$) in Fig. 2. All complex hydrogels examined were shown to exhibit non-Newtonian fluid behavior with shear thinning at an increasing shear rate. To model the stress-deformation behavior of the complex hydrogels, the Ostwald-de Waele rheological mode and Herschel-Bulkley-Papanastasiou model are described in the Supplemental file.

#### Oscillatory Frequency Sweep

**Figure 3:**
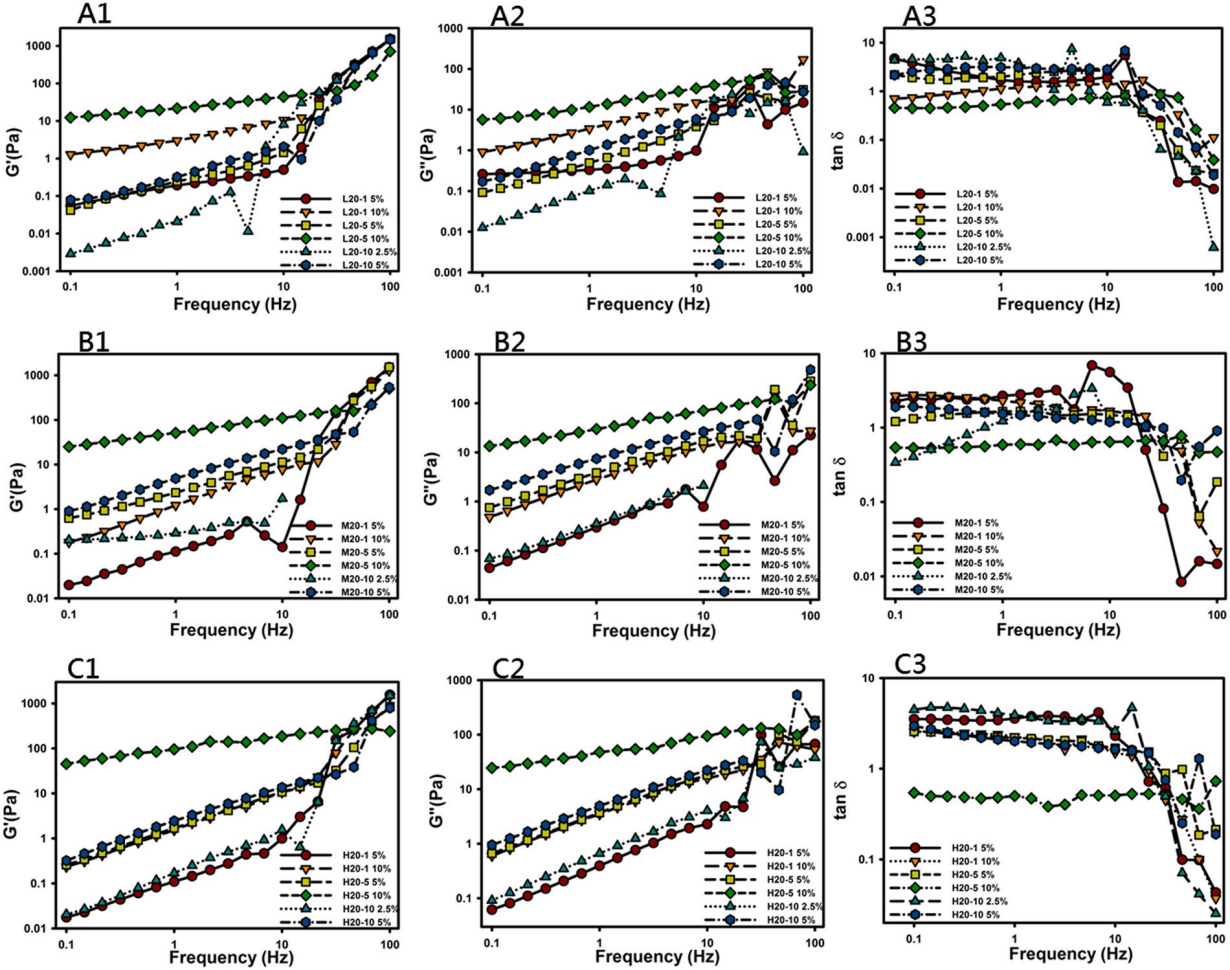
Frequency sweeps of ring-opened polyvinyl pyrrolidone (*ro*PVP)/chitosan (CS) hydrogels composed of *ro*PVP complexed with CS of low (L), medium (M), and high (H) molecular weights (Mws) at various ratios of 20:1, 20:5, and 20:10. (**A1**) L20:1; (**A2**) L20:5; (**A3**) L20:10; (**B1**) M20:1; (**B2**) M20:5; (**B3**) M20:10; (**C1**) H20:1; (**C2**) H20:5; (**C3**) H20:10.

Frequency sweep experiments were carried out to investigate the gel properties of the swollen CS/*ro*PVP complex hydrogels, namely, the stability of 3D physically cross-linked networks. The CS/*ro*PVP complex hydrogels were subjected to a frequency sweep from 0.1 to 100 Hz at an appropriate constant shear stress for each sample selected from the LVR profiles presented above (Fig. 3) to be as high as possible to avoid a torque that was too low. Results in Fig. 3 show the effects of the oscillating frequency on the elastic modulus (G′, Fig. 3A1,B1 and C1), the loss modulus (G″, Fig. 3A2,B2 and C2), and tanδ (Fig. 3A3,B3 and C3) of the complex hydrogels. The shear modulus for those complex hydrogels with a 10% concentration and a *ro*PVP/CS ratio of 20:5 (L20-5, M20-5, and H20-5) showed some frequency independence, and also the elastic modulus, G′, was dominant over the entire frequency range examined. This is indicative of a stable physically cross-linked network showing little change in viscoelastic characteristics. The shear modulus for the other complex hydrogels, on the other hand, was frequency-dependent, and in those cases, was dominated by the viscous modulus, G″, which indicates that those complex hydrogels had less cohesion in their internal network and were easily disturbed.

At higher frequencies, however, the shear moduli (G′ and G″) for those complex hydrogels with a 10% concentration and a *ro*PVP/CS ratio of 20:5 (L20-5, M20-5, and H20-5) exhibited either a gradual upturn or no increase, whereas for other complex hydrogels, an abrupt upturn was observed. This behavior was expected because at higher frequencies, polymeric chains fail to rearrange themselves in the time scale of the imposed motion and, therefore, stiffen up, assuming a more “solid-like” behavior that is characterized by an increase in G′, as observed. The longer that flexible polymeric chains are, the longer the relaxation time is that results, and the slope of the increment in G′ and G″ at higher frequencies is expected to be larger for G′. This indicates that the longer flexible polymeric chains for those complex hydrogels possessing less cohesion in their internal network as described above would cause them to show an abrupt increase in the shear modulus at higher frequencies while less-flexible polymer chains for those complex hydrogels with dominant elasticity would give rise to either a gradual increase or no increase in the shear modulus at higher frequencies.

As further shown in the plots of tanδ versus the oscillating frequency (Fig. 3A3, B3 and C3), it was observed that tanδ was frequency-independent in the frequency range of 0.1~10 Hz, whereas tanδ decreased to <1 with an increasing frequency. This is indicative of a stable cross-linked network showing little change in viscoelastic characteristics. But at higher applied frequencies (>10 Hz), the response of G′ for all complex hydrogels to the applied frequency was more significant than that for G″ resulting in the complex hydrogels stiffening up and assuming a more “solid-like” behavior that was characterized by a decrease in tanδ to <1. Since the GRDDF is expected to be subject to a frequency of contractions of three per minute in the stomach, this more “solid-like” behavior of complex hydrogels composed of *ro*PVP/CS with increasing oscillating frequency might be beneficial in terms of maintaining their integrity within the stomach.

#### Creep Compliance

When a polymer gel or other viscoelastic solid is subjected to a constant stress, *τ*, it will eventually reach a constant deformation, *γ*. The equilibrium deformation, *γ*, scaled by the applied stress, *τ*, is called the creep compliance, *J* = *γ*/*τ*, and is indicative of the equilibrium modulus of the gel, with stiffer gels deforming less (i.e., low compliance) than softer gels. Plots of creep compliance versus time for complex hydrogels are shown in Fig. S2 (Supplemental file). For those complex hydrogels composed of the same Mw of CS, the higher the polymeric concentration and CS/*ro*PVP ratio were, a lower compliance with a longer time to reach equilibrium was observed, indicating the formation of stiffer complex hydrogels with a higher polymeric concentration and a higher CS/*ro*PVP ratio. When comparisons of creep compliance were made among complex hydrogels composed of different Mws of CS at the same polymeric concentration of 5% and the same CS/roPVP ratio of 5:20 (L20-5 in Fig. S2A, M20-5 in Fig. S2B, and H20-5 in Fig. S2C), it demonstrated that the creep compliance obviously decreased with an increasing Mw of CS. The same phenomena were observed for MMw and HMw of CS at the other polymeric concentrations and different CS/roPVP ratios. These results were consistent with those described above, that the storage modulus (G′) for stiffer complex hydrogels was higher leading to lower compliance.

### Gastroretentive Properties

The influences of different Mws of CS and various *ro*PVP/CS ratios on the gastroretentive properties, including the swelling ability of the complex hydrogel tablets and the mucoadhesive ability of complex hydrogels so obtained, were evaluated.

#### Swelling Ability

**Figure 4:**
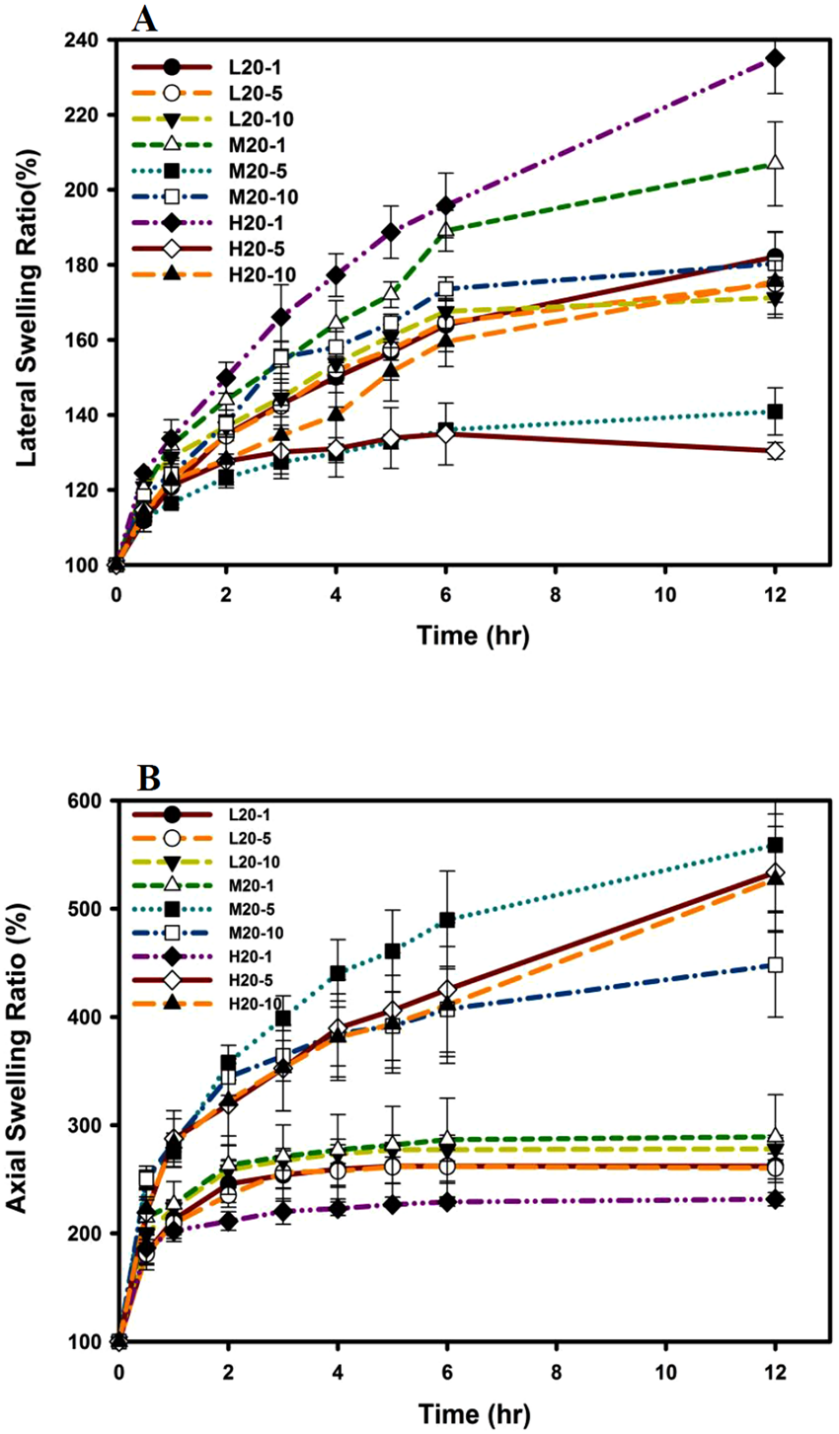
Lateral (**A**) and axial (**B**) swelling abilities of tablets prepared with ring-opened polyvinyl pyrrolidone (*ro*PVP)/chitosan (CS) hydrogels composed of *ro*PVP complexed with either low molecular weight (Mw) CS (L), medium Mw CS (M), or high Mw CS (H) at three *ro*PVP:CS ratios of 20:1 (20-1), 20:5 (20-5), and 20:10 (20-10).

For the lateral swelling ratio as shown in Fig. 4A, complex hydrogel tablets composed of complex hydrogels at a *ro*PVP/CS ratio of 20:1 (L20-1, M20-1, and H20-1) were observed to have the highest lateral swelling ratio in the order of M20-1 > H20-1 > L20-1; those composed of complex hydrogels at a *ro*PVP/CS ratio of 20/10 (L20-10, M20-10, and H20-10) were observed to have the next highest lateral swelling ratio with the same order of M20-10 > H20-10 > L20-10; and those composed of complex hydrogels at a *ro*PVP/CS ratio of 20/5 (L20-5, M20-5, and H20-5) were observed to have the smallest lateral swelling ratio but in the order of L20-5 > M20-5 > H20-5. However, the axial swelling ratio shown in Fig. 4B seems to illustrate a contrary result, of the highest axial swelling ratios being observed for those complex hydrogel tablets composed of complex hydrogels at a *ro*PVP/CS ratio of 20:5 in the order of M20-5 > H20-5 > L20-5; those composed of complex hydrogels at a *ro*PVP/CS ratio of 20:10 still had the next highest lateral swelling ratio in the order of H20-10 > M20-10 > L20-10; and those composed of complex hydrogels at a *ro*PVP/CS ratio of 20:1 were observed to have the smallest lateral swelling ratio in the order of M20-1~L20-1 > H20-1.

#### Mucoadhesive Measurements

The viscosities and mucoadhesive forces of CS, PVP, and CS/*ro*PVP complex hydrogels at various ratios are listed in Table 1. A synergetic enhancement of mucoadhesion for CS/*ro*PVP complex hydrogels was calculated and is given in parentheses. It shows that *η*_ad_ values and forces of mucoadhesion for CS/*ro*PVP increased with increasing proportions of CS in CS/*ro*PVP complex hydrogels (*p* < 0.05), and the same was true for the synergetic enhancement of mucoadhesion. A similar trend was also found for CS/*ro*PVP complex hydrogels with different Mws of CS (LMw, MMw, and HMw). Both indicated that CS/*ro*PVP complex hydrogels were able to interact more strongly with mucin to enhance the mucoadhesion of complex hydrogel tablets.

**Table 1:** Viscosity of a 9.38% (w/v) mucin solution (*η*_m_) plus 0.50% (w/v) polymer solution (*η*_p_ + *η*_m_), of the system (*η*_t_), and viscosity due to mucoadhesion (*η*_ad_) as well as the force of mucoadhesion (*F*) of blends of different molecular weights (Mws) of chitosan (CS), ring-opened polyvinyl pyrrolidone (*ro*PVP), and *ro*PVP/chitosan (CS) at various ratios at 25 °C using a shear rate of 10 per second.

Mucin + Polymer (*ro*PVP:CS=)		Viscosity (mPa)			F (dyne/cm)
		*η*_p_ + *η*_m_	*η*_t_	*η*_ad_	
1:0 (*ro*PVP)		57.20	64.10	6.90 (−)*	0.69
20:1	LMw CS	58.17	69.94	11.77 (4.38)	1.18
	MMw CS	58.94	73.95	15.01 (3.85)	1.50
	HMw CS	59.02	103.00	43.99 (32.49)	4.40
20:5	LMw CS	58.32	76.47	18.15 (9.20)	1.82
	MMw CS	58.72	107.00	48.29 (23.50)	4.83
	HMw CS	60.89	124.00	63.11 (36.90)	6.31
20:10	LMw CS	58.95	105.00	46.05 (35.73)	4.60
	MMw CS	63.21	139.00	75.79 (39.07)	7.58
	HMw CS	62.57	148.00	85.43 (46.34)	8.54
0:1 (CS)	LMw CS	59.71	76.88	17.17 (−)	1.72
	MMw CS	65.65	162.00	96.35 (−)	9.64
	HMw CS	74.51	178.00	103.49 (−)	10.35

*Values in parentheses indicate a synergetic effect of viscosity and mucoadhesion. LMw, low molecular weight; MMw, medium molecular weight; HMw, high molecular weight.

Overall, based on results obtained from both the swelling and mucoadhesive studies, CS/*ro*PVP complex hydrogels with a *ro*PVP/CS ratio of 20:1 using three different Mws of CS (L20-1, M20-1, and H20-1) were selected to incorporate the model drug of alendronate at a drug loading of 70 mg/400-mg tablet. The so-obtained tablets were subjected to *in vitro* swelling and drug release and *in vivo* bioavailability studies.

### *In Vitro* Drug Release

**Figure 5:**
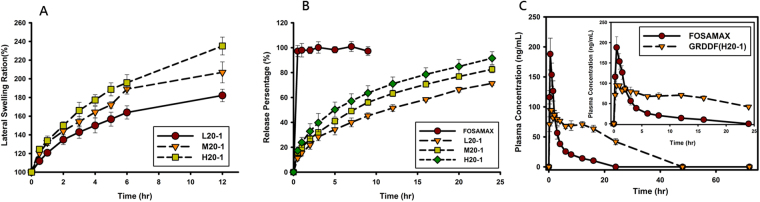
Lateral swelling abilities of (**A**) and dissolution profiles of alendronate in simulated gastric fluid (SGF) from (**B**) FOSAMAX and tablets prepared with ring-opened polyvinyl pyrrolidone (*ro*PVP)/chitosan (CS) hydrogels composed of *ro*PVP complexed with either low molecular weight (Mw) CS, medium Mw CS, or high Mw CS at a *ro*PVP:CS ratio of 20:1. (**C**) Plasma alendronate concentration profiles after administration of FOSAMAX and a gastroretentive drug delivery formulation (GRDDF) (H20-1) (mean ± SD, *n* = 3) for 72 h.

Swelling profiles and drug release profiles are respectively illustrated in Fig. 5A,B. They clearly demonstrate that the addition of alendronate to the complex hydrogel tablets did not affect the swelling characteristics of the corresponding tablets, which is shown in Fig. 4A. In comparison, the swelling ratio for H20-1 still appeared to be the greatest and fastest, followed in order by M20-1 > L20-1. Figure 5B shows that the release rate of alendronate from FOSAMAX was so much faster, that 100% was released within 0.5 h, whereas those from the three formulations of L20-1, M20-1, and H20-1 were much slower than that for FOSAMAX and were obviously in a sustained manner in the order of H20-1 > M20-1 > L20-1, which was similar to the order of the swelling ratio. This indicates that the release of alendronate from FOSAMAX was instantaneous with no controlled manner. Regarding the release rate of alendronate from the three formulations (L20-1, M20-1, and H20-1) following the order of the swelling ratio, this indicates that the increase in the gel volume with swelling made the diffusion pathway less hindered, resulting in an increase in the drug release rate. Since the H20-1 formulation showed the greatest swelling ratio and a sustained manner of drug release, it was selected as the GRDDF for the *in vivo* study to compare with the commercial FOSAMAX product.

### *In Vivo* PK Studies

Plasma concentrations after oral administration of the H20-1 GRDDF or a quarter of a FOSAMAX tablet at a dose of 17.5 mg are shown in Fig. 5C, and the PK parameters are tabulated in Table 2. Values of AUC_0→∞_, *T*_max_, and *T*_1/2_ of the H20-1 GRDDF were longer than those of FOSAMAX, while the C_max_ of the H20-1 GRDDF was lower. Compared to FOSAMAX, a sustained constant plasma concentration of alendronate was observed for the orally administered H20-1 GRDDF resulting in a 3.23-fold increase in the AUC_0→∞_.

**Table 2:** Pharmacokinetic parameters of the oral administration of two alendronate formulations with a fixed dose of 17.5 mg to rabbits (mean ± SD, *n* = 3).

Formulation	FOSAMAX Tablet	GRDDF tablet (H20-1)
Tmax (h)	0.5	0.83 ± 0.29
Cmax (ng/ml)	188.05 ± 26.51	104.83 ± 17.32
Ke (h^−1^)	0.09 ± 0.02	0.04 ± 0.01
T1/2 (h)	8.15 ± 1.89	15.50 ± 1.47
AUC0-∞(h · ng/ml)	732.99 ± 144.72	2518.91 ± 272.30
Frel (%)	100%	323.64 ± 1.88

GRDDF, gastroretentive drug dosage form; T_max_, time to the maximum concentration; C_max_, maximum concentration; K_e_, terminal elimination rate constant; T_1/2_, half-life; AUC0-∞, area under the concentration-time curve from 0 to infinity; F_rel_, relative bioavailability.

## Discussion

Optimal conditions of generating *ro*PVP to form complex hydrogels with CS were basic treatment of PVP in a 0.5-M NaOH solution at a heating temperature of 50 °C for 4 h. Results of Fig. S1B show that increasing the NaOH concentration in basic treatment of PVP increased the extent of *ro*PVP generated, but it also caused the depolymerization of PVP leading to the gel strength of the resulting complex hydrogels decreasing to an extent that the desired swollen state could not be maintained. The desirable hydrogel concentration was estimated to be approximately around 10~12% for those complex hydrogels with a *ro*PVP/CS ratio of 20:1, 7.5~10% for complex hydrogels with a *ro*PVP/CS ratio of 20:5, and 5.0~7.5% for complex hydrogels with a *ro*PVP/CS ratio of 20:10 to form a solid-like hydrogel with a higher gel strength. The desirable hydrogel concentration was observed to decrease with an increasing *ro*PVP/CS ratio, since the hydrogel strength was expected to increase with an increasing *ro*PVP/CS ratio, resulting in a lower concentration needed to behave like a viscoelastic solid hydrogel. Therefore, it confirms that complex hydrogels composed of HMw CS and a higher ratio of CS to *ro*PVP were an optimal choice for formulating GRDDFs with a desirable hydrogel strength.

Results of the swelling ability indicated that those complex hydrogel tablets with the highest lateral swelling ratio showed a lower axial swelling ratio, and vice versa; this phenomenon is visualized in Fig. S3 (Supplemental file). Since a compression force was applied axially when those complex hydrogel tablets were produced, the stress relaxation of polymeric chains causing the swelling of those complex hydrogel would be greater in the axial direction. Therefore, it was observed that the axial swelling ratio for all formulations of complex hydrogel tablets was greater than the lateral swelling ratio of the corresponding complex hydrogel tablets. Further, the swelling of polymeric chains is also caused by an osmotic effect of the polymeric matrix that is expected to increase with an increasing extent of ionic interactions between the two ionic polymeric chains. Thus, it was found that the axial swelling ratio for complex hydrogel tablets composed of complex hydrogels at *ro*PVP/CS ratios of both 20:5 and 20:10 were greater than that for complex hydrogels at a *ro*PVP/CS ratio of 20:1. But, the swelling of polymer chains reached an equilibrium at a point where the swelling (osmotic) force was just balanced by the elastic restoring (entropic) force acting in the opposite direction. This means that the stronger mechanical strength or more solid-like the complex hydrogel was, the extent of swelling was less at equilibrium. This resulted in the axial swelling ratio of those complex hydrogel tables composed of complex hydrogel at a *ro*PVP/CS of 20:5 being slightly greater than those of complex hydrogel tablets composed of complex hydrogels at a *ro*PVP/CS of 20:10.

The H20-1 GRDDF was the optimized formulation that showed the greatest swelling ratio and a sustained manner of drug release. The BA study also potentially demonstrated that the H20-1 GRDDF was maintained in the stomach to slowly release alendronate, resulting in a higher AUC, a longer half-life, and a lower C_max_. The increase in the AUC by the H20-1 GRDDF usually refers to a higher BA, which can possibly be attributed to the following reasons: (i) the GRDDF was maintained in the upper part of the GI tract, especially in the stomach, to continually release alendronate upstream of the absorption site for alendronate; and (ii) the sustained-release property of the GRDDF caused the extent of absorption via the carrier mechanism to increase compared to that of the instant release formulation resulting in a higher BA. Moreover, a lower C_max_ for the H20-1 GRDDF also indicates that the upper part of the GI tract was exposed to a lower concentration of alendronate, which potentially minimized the irritation of the mucous membrane of the GI tract by alendronate.

## Conclusions

We successfully developed a *sm*GRDDF composed of polyionic complex hydrogels of CS with *ro*PVP possessing a combination of mucoadhesive and swelling capabilities to prolong gastric retention of alendronate in the upper part of the GI tract with a sustained drug release pattern. With mucoadhesion characteristic of viscous and rigid complex hydrogels, it ensured that the dosage form adhered to the gastric mucosa. Rapid and high degrees of swelling helped delay clearance through the pyloric sphincter. Further, mucosal adhesion of CS in the complex hydrogel potentially minimized local irritation by the active ingredient (alendronate sodium) by forming a protective layer on the mucosal membrane of the stomach. Also, positive and negative charge interactions between alendronate sodium and CS decreased irritation of the upper digestive mucous. Overall, such a novel *sm*GRDDF system was able to be retained in the stomach to continually and sustainably release alendronate in the upstream portion of the stomach, and it then passed through the absorption region in the upper GI tract, resulting in enhanced BA with the expectation of enhanced therapeutic efficacy. This will potentially lead to a further reduction in the dose or an increase in the dosing interval for alendronate in clinical use. Apart from these advantages, these *sm*GRDDF systems offer PK advantages, in that a constant therapeutic level was maintained over a prolonged period of time, thus reducing fluctuations in therapeutic levels, thereby minimizing the risk of side effects.

## Materials and Methods

### Materials

CS (75~85% deacetylation) with Mws of 50~190 (LMw, low Mw), 190~310 (MMw, middle Mw), and 310~375 kDa (HMw, high Mw), acetic acid (>99.8%), calcium chloride anhydrous (CaCl_2_), heparin, pamidronate disodium, o-phthalaldehyde, and 2-mercaptoethanol were provided by Sigma-Aldrich (St. Louis, MO, USA). KOLLIDON 30 (polyvinyl pyrrolidone; PVP) was supplied by BASF (Ludwigshafen, Germany); alendronate sodium was purchased from Alcon (Bangalore, India). Mucin (product no. M2378, lot no. SLBL 3184V, mucin from porcine stomach, type II) was from Sigma-Aldrich. All other chemicals used were of reagent or pharmaceutical grade.

### Preparation of CS/Ring-opened (*ro*)PVP Complex Hydrogels

The concept of preparing complex CS/*ro*PVP hydrogels was via opposite charge interactions between the carboxylic groups of *ro*PVP and amine groups of CS. However, PVP originally underwent opening of a few pyrrolidone rings due to the synthesis conditions; thus, PVP needed to be subjected to acid-alkaline hydrolysis and heating to open more pyrrolidone rings, so that its molar equivalents of acid groups increased to an extent for opposite charge interactions with CS to form a complex hydrogel with desirable mechanical properties for gastric retention. PVP that had undergone ring-opening was designated *ro*PVP. To determine the carboxylic acid groups on *ro*PVP, 125 mg of *ro*PVP was dissolved in 10 mL of deionized water and titrated with a standard NaOH solution (0.021 N) using a pH meter as an indicator to determine the molar equivalents of acid groups^33^. In this study, the influences of the following formulation and process factors on the formation of CS/*ro*PVP complex hydrogels with different Mws of CS were evaluated.

### Effects of Heating Times on the Generation of *ro*PVP to Form CS/*ro*PVP Complex Hydrogels

To assess the effects of heating times, 10 g of PVP was dissolved in 0.15 M NaOH solutions and subsequently treated with no heating or heating in a 100 °C water bath for 1 or 8 h. After treatment, 0.5 g CS of three different Mws (LMw, MMw, and HMw) dissolved in 1% acetic acid was added with stirring to form complex hydrogels. Then, nine samples were separately dialyzed (Mw cutoff of 6000~8000) against deionized water until the dialyses became neutral, and then freeze-dried. The lyophilized sample was crushed and passed through a 60-mesh sieve to obtain CS/*ro*PVP complex hydrogels. Rheological characteristics of the CS/*ro*PVP complex hydrogels dissolved in simulated gastric fluid (SGF) were evaluated. The 400-mg CS/*ro*PVP complex hydrogel tablets composed of *ro*PVP prepared by different heating times with either LMw CS, MMw CS, or HMw CS at a *ro*PVP:CS ratio of 20:1 were compressed on a Carver Laboratory Press tableting machine, without the addition of a lubricant, using a flat-faced punch and die (12 mm in diameter), for 6 s at a 1-ton compression pressure. The swelling profile of the obtained tablets in SGF was examined as an indication of the formation of CS/*ro*PVP complex hydrogels.

### Effects of NaOH Concentrations on the Generation of *ro*PVP for Forming CS/*ro*PVP Complex Hydrogels

To examine the effect of the NaOH concentration, 10 g of PVP was dissolved in various concentrations of NaOH aqueous solutions (0.5, 1.0, and 1.5 M) and then subjected to heating in a 50 °C water bath for 4 h. After treatment, 0.5 g HMw CS (310~375 kDa) dissolved in 1% acetic acid was added with stirring to form complex hydrogels. The subsequent process to prepare lyophilized powder and CS/*ro*PVP complex hydrogel tablets followed the same procedure as described above in the previous section. Rheological characteristics of the CS/*ro*PVP complex hydrogels and the swelling profile of tablets in SGF were examined.

### Effects of the CS Mw and *ro*PVP/CS Ratio on the Formation of CS/*ro*PVP Complex Hydrogels

To evaluate the effect of different Mws of CS and *ro*PVP/CS ratios, 10 g of PVP was dissolved in 0.5 M NaOH aqueous solutions and then subjected to heating in a 50 °C water bath for 4 h. After treatment, CS of three different Mws (LMw, MMw, and HMw) at three ratios of *ro*PVP/CS (20:1, 20:5, and 20:10) was dissolved in 1% acetic acid and added with stirring to form complex hydrogels. The subsequent process to prepare the lyophilized powder and complex CS/*ro*PVP tablets for nine samples followed the same procedure as described above in the previous section. Rheological characteristics of the lyophilized powder and swelling profiles of tablets in SGF were examined.

### Measurements of Rheological Properties

Rheological studies were performed using different concentrations for different ratios of *ro*PVP/CS (5% and 10% for 20:1 and 20:5; and 2.5% and 5.0% for 20:10). Rheological parameters were measured with a HAAKE Rotational Rheometer RS-1 (C60/1) (Thermo Fisher Scientific, Waltham, MA, USA). Details are described in the Supplemental file.

### Determination of the Swelling Profile

Swelling studies were conducted using a Vankel dissolution apparatus. The 400-mg CS/*ro*PVP complex hydrogel tablets composed of *ro*PVP with CS of three different Mws at three ratios of *ro*PVP:CS (20:1, 20:5, and 20:10) were immersed in 900 mL of SGF without pepsin (pH 1.2) at 37.0 ± 0.5 °C and 100 rpm. At predetermined time points (0.5, 1, 2, 3, 4, 5, 6, and 12 h), the swollen tablets were removed from the solution and immediately wiped with a paper towel to remove surface droplets. Both the lateral and axial dimensions of the swollen tablet were measured using dial calipers. Baumgartner *et al*. concluded that swelling in the axial direction was more pronounced than in the lateral direction, because compression was applied on the systems in the axial direction^34^. The swelling ratio (*S*_*r*_) for both lateral and axial directions was calculated according to the following equations:

1 $${\rm{Lateral}}\,{\rm{Swelling}}\,{\rm{Ratio}}\,({S}_{r})=({D}_{t}-{D}_{i})/{D}_{i}\,{\rm{and}}$$

2 $${\rm{Axial}}\,{\rm{Swelling}}\,{\rm{Ratio}}\,({S}_{r})=({T}_{t}-{T}_{i})/{T}_{i};$$

where *D*_*i*_ or *T*_*i*_ and *D*_*t*_ or *T*_*t*_ represent the initial diameter (*D*) or thickness (*T*) of the dry tablet and that of the swollen tablet at time *t*, respectively. The data represent the mean ± SD from at least three samples per formulation.

### Determination of Mucoadhesion by Viscosity Measurements

Viscosity measurements were performed to determine the mucoadhesion as previously described^30,35^. Theoretically, interactions between polymers (CS, *ro*PVP, and CS/*ro*PVP hydrogels) and mucin resulted in viscosity changes; these changes can be converted into mechanical energy or work. Details are described in the Supplemental file.

### *In Vitro* Drug Release Studies

Drug release from FOSAMAX and GRDDF tablets (70 mg alendronate and 330 mg CS/*ro*PVP complex hydrogels composed of *ro*PVP with either LMw CS, MMw CS, or HMw CS at a *ro*PVP:CS ratio of 20:1, flat-faced tablet, 12 mm in diameter) was determined in 900 mL of SGF at 37 ± 0.5 °C and 50 rpm, for 24 h, based on the apparatus II method (USP XXIX) (VK7020, Vankel, UK). The medium was removed at predetermined time points (0, 0.5, 1, 2, 3, 5, 8, 12, 16, and 24 h) and replaced with fresh medium of the same volume. The method to determine the amount of alendronate by an ultraviolet/visible spectrophotometer (V-550, Jasco, Tokyo, Japan) is described in the Supplemental file. The average percentage of drug dissolved at each sampling time was calculated after correcting for the cumulative amount removed in previous samples.

### *In Vivo* Pharmacokinetic (PK) Studies

All animal experiments were carried out in accordance with a protocol approved by the Laboratory Animal Center of Taipei Medical University (approval no.: LAC-2013-0126) and conducted in compliance with the Animal Welfare Act. New Zealand white rabbits weighing 4~5 kg were purchased to investigate the PK profile of alendronate GRDDF tablets compared to FOSAMAX. Rabbits were orally given a single GRDDF alendronate tablet (17.5 mg alendronate and 150 mg CS/*ro*PVP complex hydrogel composed of *ro*PVP with HMw CS at a *ro*PVP:CS ratio of 20:1 (H20-1), flat-faced tablet, 6 mm in diameter) or a quarter of a FOSAMAX tablet (17.5 mg/quarter tablet). Blood samples were collected into heparinized tubes from the right ear vein at predosing and at 0.25, 0.5, 1, 1.5, 2, 3, 4, 6, 8, 12, 16, 24, 48, and 72 h after administration. All blood samples were immediately centrifuged at 3000 rpm for 15 min at 4 °C to obtain plasma. Plasma samples were stored at −80 °C before the high-performance liquid chromatographic (HPLC) analysis (details described in the Supplemental file). PK parameters are presented as the mean and standard deviation (SD) from individual rabbits in each group and were estimated through a noncompartmental analysis. The terminal elimination rate constant (K_e_) was estimated from the slope of the log-linear phase of declining plasma concentrations of an alendronate vs. time graph. The half-life (T_1/2_) was calculated using the following equation: T_1/2_ = ln2/*K*_e_. The area under the concentration-time curve from beginning to the last time point (AUC_0→last_) was calculated using the trapezoidal method. Summation of AUC_0→last_ and the concentration at the last measured point divided by *K*_e_ yielded AUC_0→∞_. Clearance (CL) was calculated by dividing the dose by AUC_0→∞_, and the volume of distribution (V) by dividing CL by *K*_e_.

### Statistical Analysis

All results are presented as the mean ± SD. A one-way analysis of variance (ANOVA) was used to determine statistical significance (PASW Statistics 18.0). A value of *p* < 0.05 was considered statistically significant.

## Electronic supplementary material

Supplementary Information
