# Supplementary material for: Complex Hydrogels Composed of Chitosan with Ring-opened Polyvinyl Pyrrolidone as a Gastroretentive Drug Dosage Form to Enhance the Bioavailability of Bisphosphonates
Source: Sci Rep. 2018 May 25;8:8092. doi: 10.1038/s41598-018-26432-2 (PMC5970250; doi:10.1038/s41598-018-26432-2)
Supplement: Supplementary file 1 — Supplementary Information [file 41598_2018_26432_MOESM1_ESM.pdf]

Complex Hydrogels Composed of Chitosan with Ring-opened Polyvinyl Pyrrolidone as a Gastroretentive Drug Dosage Form to Enhance the Bioavailability of Bisphosphonates

Chia-Yu Su[a#], Hsiu-O Ho[a#], Ying-Chen Chen[a], Yu-Ting Yu[a], Der-Zen Liu[b], Fang-Ching Chao[a], and Ming-Thau Sheu[a,c]*

[a]School of Pharmacy, College of Pharmacy, Taipei Medical University, Taipei, Taiwan, ROC; [b]Graduate Institute of Biomedical Materials and Tissue Engineering, College of Biomedical Engineering, Taipei Medical University, Taipei, Taiwan, ROC; [c]Clinical Research Center and Traditional Herbal Medicine Research Center, Taipei Medical University Hospital, Taipei, Taiwan, ROC

Author e-mail addresses: alice5230419@hotmail.com , hsiuoho@tmu.edu.tw , domyself330@yahoo.com.tw , b303097040@tmu.edu.tw , tonyliu@tmu.edu.tw , m301105019@tmu.edu.tw , mingsheu@tmu.edu.tw

[#]The first two authors contributed equally to this work.
Corresponding author: Dr. Ming-Thau Sheu
Address: 250 Wu-Hsing Street, Taipei 110, Taipei, Taiwan, ROC
E-mail: mingsheu@tmu.edu.tw
Telephone and Fax: 886-2-23771942

Materials and methods

Measurements of Rheological Properties

We used the oscillatory shear stress and dynamic temperature ramp test methods. The linear viscoelastic region (LVR) of these hydrogels was determined, and elastic modulus (G') and viscous modulus (G'') values were measured under the same physical conditions at a constant frequency (1 Hz), while increasing the shear stress from 0.003 to 200 Pa and holding the temperature at 25±2 ℃. Oscillation frequency sweep measurements were performed at a frequency range of 0.1~100 Hz with a constant stress selected from the corresponding LVR range of these hydrogels at a constant temperature of 25±2 ℃. The main rheological technique to characterize hydrogels is small-amplitude oscillatory shear (SAOS), which is meant to be carried out within the linear viscoelastic region of a material, ensuring the measured hydrogel properties are independent of the magnitude of the imposed strain or stress. In SAOS measurements, the shear storage modulus, G', loss modulus, G'', and loss factor, tanδ, are critical hydrogel properties monitored against time, frequency, and strain. G' measures the deformation energy stored during the shear process of a test material (i.e., the stiffness of the material), and G'' is representative of the energy dissipated during shear (i.e., the flow or liquid-like response of the material). If G'' > G' ($\tan\delta = G''/G' > 1$), the sample behaves more like a viscoelastic liquid, while conversely,

when G' > G", and, thus, tanδ < 1, the sample has the properties of a viscoelastic solid. To model the stress-deformation behavior of the complex hydrogels dominated by a more liquid-like flow pattern, the Ostwald-de Waele rheological model or power law model (Eq. 1) was selected to account for the shear thinning behavior without the existence of yield stress (τ_y) while the Herschel-Bulkley-Papanastasiou model (Eq. 2) was used for the shear thinning behavior with the existence of yield stress (τ_y) and the exponential growth of the yield stress (τ_y) regularized by m at very low applied shear rates [1].

$$\tau = K_c \dot{\gamma}^n \qquad (1)$$
$$\tau = \tau_y [1 - EXP(-m\dot{\gamma})] + K_c \dot{\gamma}^n (2)$$
$$\tau = \tau_y + K_c \dot{\gamma}^n \qquad (3)$$

In these equations, τ is the shear stress, $\dot{\gamma}$ is the shear rate, τ_y is the yield stress, K_c is the consistency index indicating the resistance offered by a real fluid to such deformation, and n is the power law index measuring the degree of departure from Newtonian behavior. When the value of n is < 1, the type of flow is shear thinning (pseudoplastic behavior). The type of flow is shear thickening (dilatant behavior) when n is > 1. As n approaches 1, the flow becomes less shear dependent, and an n value equal to 1 indicates Newtonian flow. The exponential regularization parameter, m, was introduced in the Herschel-Bulkley-Papanastasiou model, which controls the exponential growth of the yield stress and has dimensions of time. The exponent m

controls the stress growth, such that below the yield stress, τ_y, a finite stress is allowed for vanishingly small shear rates, while beyond the yield stress, the growth of the stress with the shear rate follows the Herschel-Bulkley model as shown by Equation 3. These three Herschel-Bulkley parameters of τ_y, K_c, and n, were determined experimentally to characterize the stress-deformation behavior. A common way of dealing with the non-linearity associated with the Herschel-Bulkley model is to estimate the yield stress, τ_y, and then find the other two parameters using linear techniques. An alternative approach proposed by Glen Mullineux to avoid the shortcoming of the highly sensitive K_c and n to the estimate of the yield stress, τ_y, was adopted to obtain these three parameters in this study [2]. Making use of the particular form of the Herschel-Bulkley model, a function $F(n)$ was obtained in which the flow behavior index, n, is a single well-defined root (other than at the origin). This allows n to easily be determined by successive bisection, and then it is straightforward to determine the other unknown parameters by a linear regression of Equation 2 with known value of n. Since only those shear stresses larger than the yield stress comply with the Herschel-Bulkley model, the evaluation of rheological data based on the Herschel-Bulkley equation (Eq. 3) to obtain three Herschel-Bulkley parameters of τ_y, K_c, and n was compared by utilizing different ranges of the dataset, one utilizing the entire range of the dataset (shear rate: 0.1~1000 s^{-1}) and the other utilizing a partial

range of the dataset beyond the yield stress (shear stress: 100~1000 s^{-1}). The Ostwald-de Waele rheological model or power law model that signifies no existence of yield stress in the rheograms was also included to evaluate the consistency index, K_c, and the power law index, n, of these complex hydrogels for comparison with the Herschel-Bulkley model that signifies the existence of yield stress. Values of K_c and n of all samples were determined by the least squares regression method.

Determination of Mucoadhesion by Viscosity Measurements

The 2% CS solution or CS/*ro*PVP hydrogels solution were prepared by dissolving in 2% hydrochloric acid (HCl). The 2% *ro*PVP solution was prepared by dissolving in deionized water. Mucin solutions at concentrations of 2% (w/v) and 15% (w/v) were prepared by dispersing the appropriate amount of mucin in deionized water. For viscosity measurements, 5 mL of mucin solution (15%, w/v) was mixed with 2 mL of (2%, w/v) polymer (CS, *ro*PVP, and CS/*ro*PVP hydrogels). The final volume of the mixtures was adjusted to 8 mL with deionized water (the pH was adjusted to 4.5), and the mixtures were mixed until homogeneous. The final concentrations of polymer and mucin solutions were 0.50% (w/v) and 9.38% (w/v), respectively. These mixtures of mucin–polymers were equilibrated at 25.0 ± 0.1℃ for 1 h before viscosity measurements. The viscosity component caused by

mucoadhesion (η_{ad}) can be calculated according to Equation 4:

$$\eta_{ad} = \eta_s - \eta_m - \eta_p; \quad (4)$$

where η_s is the viscosity of the system, and η_m and η_p are the viscosities of pure mucin and polymer, respectively. All viscosity values were measured at the same temperature and rate of shear. Subsequently, the force of mucoadhesion (F_m) was determined using Equation 5:

$$F_m = \eta_{ad} \times \sigma; \quad (5)$$

where σ is the shear rate (per second).

UV method for alendronate

A method to determine the amount of alendronate by the formation of the complex between non-chromophoric compound, alendronate, and iron (III) chloride in a perchloric acid solution developed by Kuljanin et al. was utilized for analysis of the alendronate concentration in the dissolution medium [3]. The concentration of the complex was measured by an ultraviolet/visible spectrophotometer (V-550, Jasco, Tokyo, Japan) at a wavelength of 300 nm, using a 10-mm quartz cell. The method was validated to have acceptable precision (0.16%~7.20% and 0.44%~9.33%, respectively) and accuracy (-3.72%~3.95% and -1.51%~4.28%, respectively) for inter- and intra-day assays.

HPLC method for alendronate

A simple and rapid HPLC method for quantifying alendronate in plasma reported by Meng et al. was utilized with a minor modification [4]. Plasma samples stored in the freezer at -80 ℃ were allowed to thaw at room temperature before processing. The internal standard solution (3 μg/mL pamidronate; 20 μL) and 20 μL of 0.05 M NaOH were added to 200 μL of plasma and then the tube was briefly shaken. After dilution with 1 mL water, 20 μL of 0.1 M $CaCl_2$ and the same volume of 0.1 M K_2HPO_4 were added, and the sample was made alkaline with 40 μL of 1 M NaOH. The sample was centrifuged for 20 min at 6000 rpm (2000 *g*) and 4 ℃, and the supernatant was discarded. The precipitate was completely reconstituted in 40 μL of 0.2 M HAc, 20 μL of 0.1 M EDTA-Na_2, and 40 μL of 0.2 M NaAc by vortex-mixing for 6 min. One hundred microliters of the resulting solution was transferred to an HPLC vial, then 20 μL of the OPA/2ME reagent (freshly prepared by dissolving 12.5 mg of anhydrous o-phthalaldehyde (OPA) with 2 mL of ethanol, then 60 μL of 2-mercaptoethanol (2ME) solution was added, and the volume was made up to 50 mL with 0.05 M NaOH) and 12 μL of 1.0 M NaOH were added and vortexed for few seconds. From the resulting solution, 50 μL was immediately injected for the HPLC analysis.

HPLC separation was achieved on a column of Atlantic® T3 C_{18} (250 × 4.6 mm, 5 μm, Waters, Milford, MA, USA) maintained at a temperature of 40 ℃ using

acetonitrile-0.4% EDTA-Na_2 (16:84, v/v) containing 0.034% of NaOH as the mobile phase pumped at a flow rate of 1 ml/min (PU-980, Jasco). The fluorometric detector (FP2020, Jasco) was operated at 339 nm (excitation) and 447 nm (emission). The linearity over the concentration range of 7.5~300 ng/mL for alendronate was obtained, and the lower limit of quantification was 7.5 ng/mL. This analytical method was validated with inter-day and intra-day precisions within 2.40%~8.34% and 0.57%~11.39%, respectively, and accuracies of -2.53%~5.52% and -5.34%~1.33%, respectively.

Results

To model the stress-deformation behavior of the complex hydrogels

Table S1 lists the comparison of two power law parameters of K_c (the consistency index or dynamic viscosity) and *n* (the flow index) evaluated based on the Ostwald-de Waele rheological model (**Eq. 1**) and three Herschel-Bulkley parameters of τ_y, K_c, and *n* evaluated based on the Herschel-Bulkley equation (**Eq. 2**) but one utilizing the entire range of the dataset (0.1~1000 s^{-1}, **Table S2,** and the other utilizing a partial range of the dataset (100~1000 s^{-1}, **Table S3**). It demonstrates that regression coefficients for the Herschel-Bulkley equation (**Eq. 2**) utilizing a partial range of the dataset (100~1000 s^{-1}) were among the best, showing that the Herschel-Bulkley model is more appropriate to describe the flow behavior of these complex hydrogels. The negative yield stress values observed for those complex hydrogels indicated that they behaved more liquid-like. However, negative yield stress values were found to be close to 0, indicating the yield stress, τ_y, might not exist, and therefore those complex hydrogels behaved more liquid-like. On the other hand, the yield stress was observed to be positive for complex hydrogels that behaved more solid-like. This indicates that such a complex hydrogel at rest consists of 3D structures of sufficient rigidity to resist any external stress less than the yield stress, τ_y, and therefore offers enormous resistance to flow. In contrast, for stress levels above the yield stress, τ_y, however, the

structure breaks down and the substance behaves like a viscous material. For cases of polymeric hydrogel systems, microstructural units composed of entangled polymeric chains are randomly oriented corresponding to their minimum energy state at rest. At low levels of shear, the system resists any deformation by offering a very high resistance either by exhibiting a very high value of viscosity or a yield stress. As the magnitude of the external shear stress gradually increases, the structural units (also known as ”flow units”) respond by aligning themselves with the direction of flow, or by deforming to orient along the streamlines. Polymer molecules which are coiled and entangled at low shear rates gradually become disentangled, and finally fully straighten out. All these microstructural changes facilitate flow, i.e., they lead to a lowering of the apparent viscosity with shear which leads to shear-thinning behavior.

Since the flow index, *n,* is also called the fluidity index, the more shear thinning that is produced, the lower and the closer is the value of *n* to 0. The consistency index, K_c, represents the overall range of viscosities across the part of the flow curve that is modeled, and it also can be referred to as the dynamic viscosity of a viscoelastic hydrogel characterized by G''/ω (loss modulus/angular frequency). Therefore, the K_c value expressed by the liquid-like component for those viscoelastic hydrogels dominantly behaving solid-like would decrease with an increasing solid-like behavior,

whereas the K_c value for those viscoelastic hydrogels dominantly behaving liquid-like would become larger with increasing liquid-like behavior. With greater liquid-like components, the extent of shear thinning increases resulting in the value of *n* value being lower and closer to 0. As shown in **Table S3**, increases in the K_c value with an increasing polymer concentration, Mw of CS, and *ro*PVP/CS ratio were observed for those viscoelastic hydrogels behaving dominantly liquid-like, except for those behaving dominantly solid-like of L20-5, M20-5, and H20-5 at a 10% polymer concentration, which shows the decrease in the K_c value with an increase in the Mw of CS. Correspondingly, the *n* value was observed to be smaller for those more liquid-like viscoelastic hydrogels with a larger value of K_c, indicating that the greater the liquid-like components, the more shear thinning that results. On the other hand, the *n* value was observed to be larger for those more solid-like viscoelastic hydrogels with a smaller value of K_c indicating that the more solid-like behavior there is, the less shear thinning that results.

Table S1. Comparisons of regression parameters (τ, K_c, n, and regression coefficient) based on the Power law equation for various concentrations of ring-opened polyvinyl pyrrolidone (*ro*PVP)/chitosan (CS) complex hydrogels composed of different Mw CS (L, low Mw; M, medium Mw; and H, high Mw) at three different ratios (20:1, 20:5, 20:10).

	**20-1 (5%)**	**20-1 (10%)**	**20-5 (5%)**	**20-5 (10%)**	**20-10 (2.5%)**	**20-10 (5%)**
			K_c			
**LMw**	0.125	1.658	0.171	49.408	0.046	0.391
**MMw**	0.131	1.729	2.407	24.900	0.136	5.150
**HMw**	0.190	1.969	2.095	21.468	0.274	4.616
			n			
**LMw**	0.725	0.592	0.783	0.308	0.799	0.750
**MMw**	0.749	0.618	0.530	0.436	0.762	0.510
**HMw**	0.739	0.640	0.584	0.475	0.717	0.585
			R^2			
**LMw**	0.9987	0.9863	0.9987	0.9880	0.9874	0.9995
**MMw**	0.9985	0.9868	0.9677	0.9405	0.9977	0.9589
**HMw**	0.9985	0.9863	0.9796	0.9673	0.9965	0.9780

$\tau = K_c \gamma^n$, where τ is shear stress, *g* shear rate, K_c consistency index, and *n* the power law index or flow behavior index.

Table S2. Comparisons of regression parameters (τ, K_c, n, and regression coefficient) based on Herschel-Bulkley model with full range of shear rate data (0.1-1000 S^{-1}) for various concentrations of ring-opened polyvinyl pyrrolidone (*ro*PVP)/chitosan (CS) complex hydrogels composed of different Mw CS (L, low Mw; M, medium Mw; and H, high Mw) at three different ratios (20:1, 20:5, 20:10).

	**20-1 (5%)**	**20-1 (10%)**	**20-5 (5%)**	**20-5 (10%)**	**20-10 (2.5%)**	**20-10 (5%)**
			Yield stress (τ_0, Pa)			
**LMw**	-0.65	3.24	-0.95	-123.04	-0.03	-1.06
**MMw**	-0.69	3.68	-6.38	-6.16	-1.08	3.30
**HMw**	-0.97	3.41	-4.27	26.94	-1.55	7.97
			K_c			
**LMw**	0.236	1.251	0.310	122.960	0.033	0.512
**MMw**	0.245	1.271	5.736	37.745	0.314	5.625
**HMw**	0.326	1.686	4.141	18.438	0.607	3.406
			n			
**LMw**	0.633	0.628	0.695	0.212	0.854	0.711
**MMw**	0.657	0.659	0.405	0.371	0.640	0.490
**HMw**	0.661	0.658	0.482	0.487	0.600	0.625
			R^2			
**LMw**	0.9996	0.9981	0.9998	0.9930	0.9999	0.9999
**MMw**	0.9997	0.9983	0.9980	0.9918	0.9994	0.9946
**HMw**	0.9995	0.9988	0.9998	0.9885	0.9997	0.9968

$\tau = \tau_0 + K_c \gamma^n$, where τ is shear stress, τ_0 yield stress, γ shear rate, K_c consistency index, and n the power law index or flow behavior index.

Table S3. Comparisons of regression parameters (τ, K_c, n, and regression coefficient) based on Herschel-Bulkley model with partial range of shear rate data (100-1000 S^{-1}) for various concentrations of ring-opened polyvinyl pyrrolidone (*ro*PVP)/chitosan (CS) complex hydrogels composed of different Mw CS (L, low Mw; M, medium Mw; and H, high Mw) at three different ratios (20:1, 20:5, 20:10)

	**20-1 (5%)**	**20-1 (10%)**	**20-5 (5%)**	**20-5 (10%)**	**20-10 (2.5%)**	**20-10 (5%)**
			Yield stress (τ_0, Pa)			
**LMw**	-0.15	0.14	-0.24	0.16	-0.04	-0.32
**MMw**	-0.15	-0.41	-0.78	4.77	-0.26	-1.26
**HMw**	-0.25	-0.53	-0.29	8.76	-0.25	-2.12
			K_c			
**LMw**	0.184	1.563	0.254	50.969	0.033	0.454
**MMw**	0.195	1.568	3.993	33.976	0.232	5.985
**HMw**	0.259	1.963	3.301	23.251	0.456	4.237
			n			
**LMw**	0.666	0.600	0.722	0.302	0.855	0.727
**MMw**	0.687	0.633	0.449	0.383	0.680	0.485
**HMw**	0.692	0.639	0.510	0.458	0.637	0.599
			R^2			
**LMw**	0.9992	0.9980	0.9996	0.9901	0.9999	0.9998
**MMw**	0.9995	0.9994	0.9956	0.9874	0.9988	0.9968
**HMw**	0.9992	0.9995	0.9995	0.9813	0.9994	0.9985

$\tau = \tau_0 + K_c \gamma^n$, where τ is shear stress, τ_0 yield stress, γ shear rate, K_c consistency index, and n the power law index or flow behavior index.

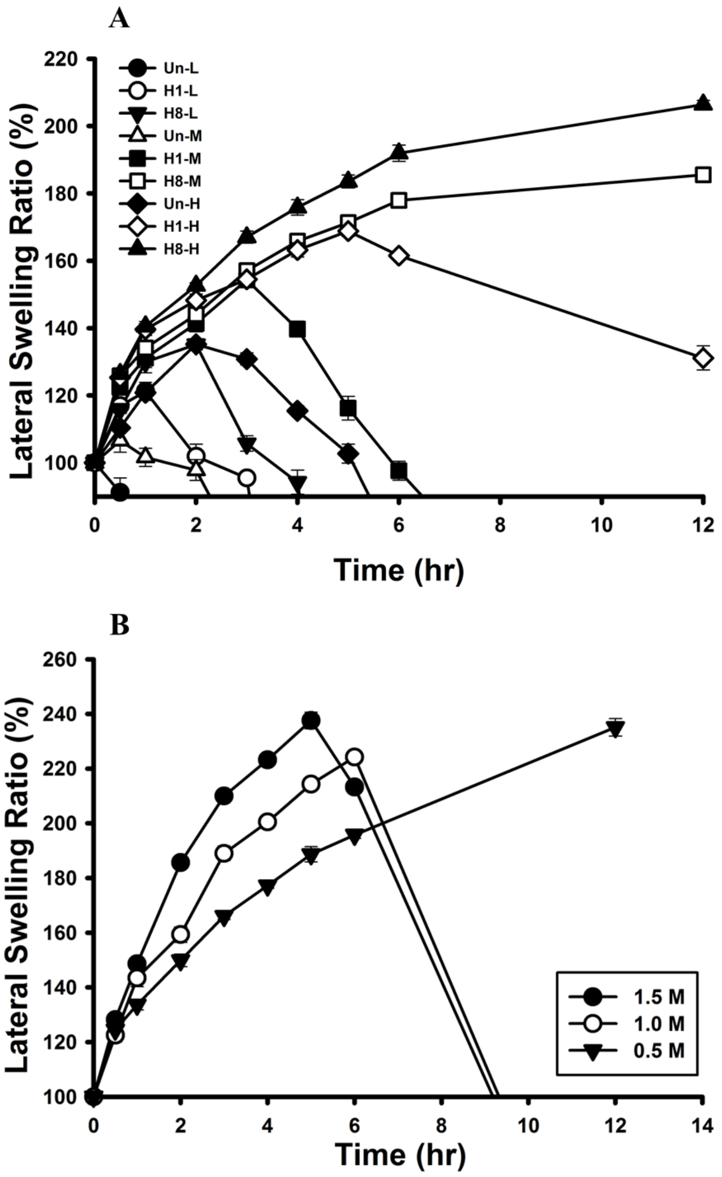

Figure S1. Swelling ability of various ring-opened polyvinyl pyrrolidone (*ro*PVP)/chitosan (CS) hydrogels composed of *ro*PVP prepared by treatment with a 0.15 M NaOH solution for different heating times and complexes with chitosan of different Mws (Un, no heating; H1, 1 h heating; H8, 8 h heating; Low/Med/High indicate the Mw of CS) (*n*=3). **B:** Swelling ability of *ro*PVP/CS hydrogels composed of *ro*PVP prepared by treatment with a NaOH solution of different concentrations at 50 °C with heating for 4 h and complexes with CS of high Mw at a 20:1 ratio. (*n*=3).

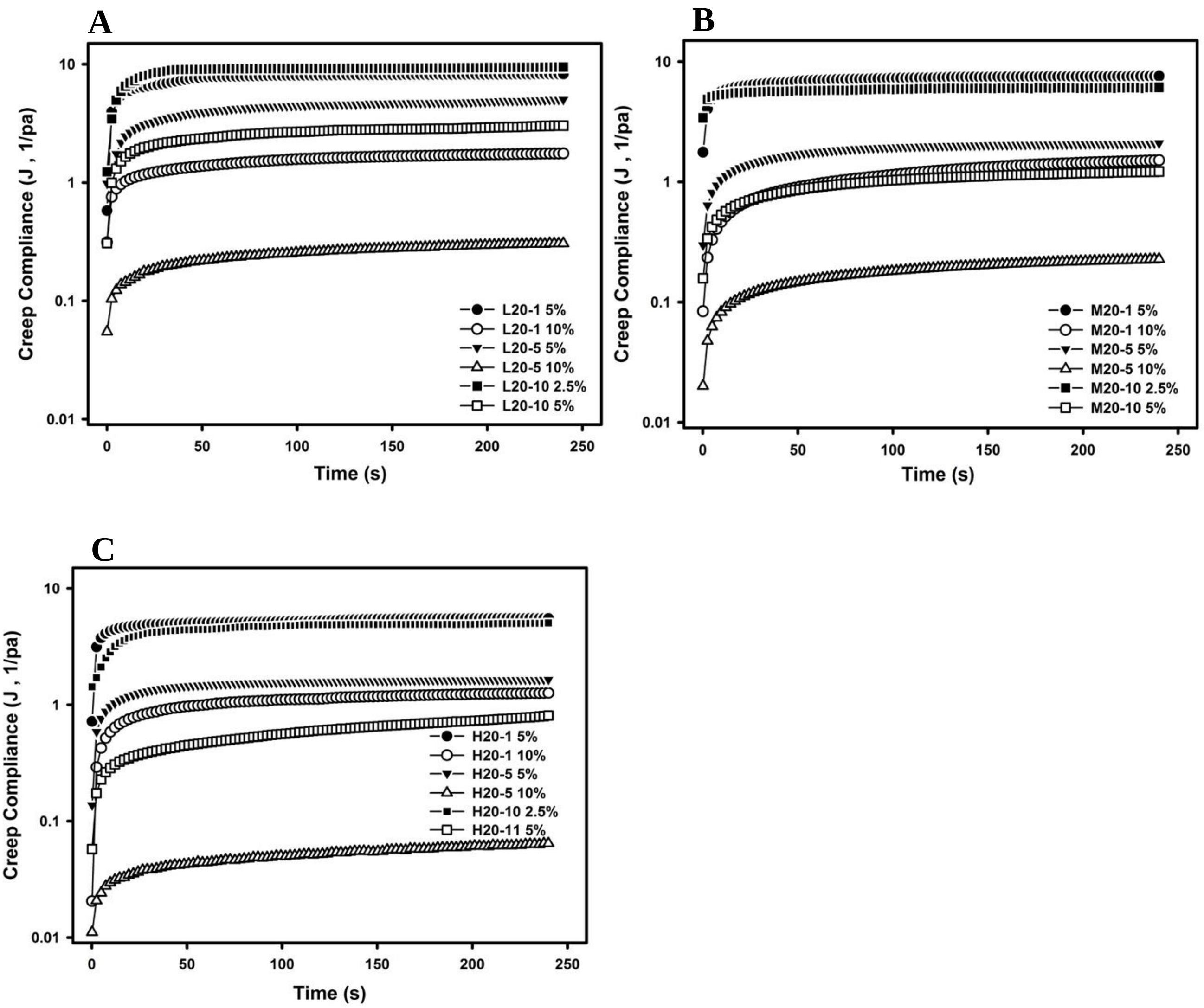

Figure S2. Creep compliance of ring-opened polyvinyl pyrrolidone (*ro*PVP)/chitosan (CS) hydrogels composed of *ro*PVP complexed with either low Mw CS (**A1**), medium Mw CS (**A2**), or high Mw CS (**A3**) at three *ro*PVP:CS ratios of 20:1, 20:5, and 20:10 or with different Mws of CS at the same *ro*PVP:CS ratios of 20:1 (**B1**), 20:5 (**B2**), 20:10 (**B3**).

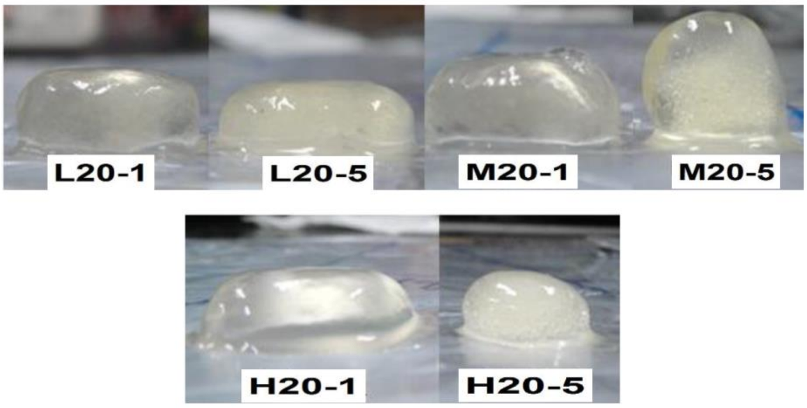

Figure S3. Photographs of tablets prepared with ring-opened polyvinyl pyrrolidone (*ro*PVP)/chitosan (CS) hydrogels composed of *ro*PVP complexed with either low Mw CS, medium Mw CS, or high Mw CS at two *ro*PVP:CS ratios of 20:1 and 20:5 after swelling 12 h in simulated gastric fluid (SGF).

Reference

1 Mitsoulis, E. *Flows of Viscoplastic Materials: Models and Computations*. Vol. 135 135-178 (Rheology reviews, 2007).

2 Mullineux, G. Non-linear least squares fitting of coefficients in the Herschel–Bulkley model. *Applied Mathematical Modelling* **32**, 2538-2551, doi:doi.org/10.1016/j.apm.2007.09.010 (2008).

3 Kuljanin, J., Janković, I., Nedeljković, J., Prstojević, D. & Marinković, V. Spectrophotometric determination of alendronate in pharmaceutical formulations via complex formation with Fe(III) ions. *Journal of Pharmaceutical and Biomedical Analysis* **28**, 1215-1220, doi:10.1016/S0731-7085(02)00021-3 (2002).

4 Meng, J., Meng, Q. & Zheng, L. A simple and rapid high-performance liquid chromatography method for determination of alendronate sodium in beagle dog plasma with application to preclinical pharmacokinetic study. *Biomed Chromatogr* **24**, 169-173, doi:10.1002/bmc.1266 (2010).
